# Activity of Sulbactam-Durlobactam and Comparators Against a National Collection of Carbapenem-Resistant *Acinetobacter baumannii* Isolates From Greece

**DOI:** 10.3389/fcimb.2021.814530

**Published:** 2022-01-20

**Authors:** Dimitra Petropoulou, Maria Siopi, Sophia Vourli, Spyros Pournaras

**Affiliations:** Laboratory of Clinical Microbiology, Attikon University Hospital, School of Medicine, National and Kapodistrian University of Athens, Athens, Greece

**Keywords:** hospital infections, diazabicyclooctane, durlobactam, carbapenemases, beta-lactamase inhibitor, serine-type beta-lactamases, CRAB infections, sulbactam-durlobactam-imipenem

## Abstract

**Background:**

*Acinetobacter baumannii* is a leading cause of healthcare-associated infections worldwide, due to both its persistence in the hospital setting and ability to acquire high levels of antibiotic resistance. Carbapenem-resistant *A. baumannii* isolates (CRAB) limit the activity of current antimicrobial regimens and new alternatives or adjuncts to traditional antibiotics are urgently needed. Durlobactam is a novel broad-spectrum inhibitor of serine-type β-lactamases that restores sulbactam (SUL) activity against *A. baumannii*. The sulbactam-durlobactam (SD) combination has recently completed Phase 3 testing in the global ATTACK trial.

**Objectives:**

The aim of this study is to evaluate the *in vitro* activity of SD versus comparators against a representative nationwide collection of CRAB isolates.

**Methods:**

One hundred ninety CRAB isolates were collected from clinical samples of patients hospitalized in 11 hospitals throughout Greece during 2015. *In vitro* activities of SD and comparators (SUL alone, amikacin, minocycline, imipenem, meropenem, colistin, SD and imipenem combined with SD) were determined by broth microdilution.

**Results:**

Durlobactam restored sulbactam activity against the majority of the strains tested, with SD exhibiting the lowest MIC_90_ (8 μg/ml) relative to the other single comparators tested; 87.9% of the isolates had SD MICs ≤4/4 µg/ml. The most active comparator was colistin (MIC_90_ = 16 μg/ml). The addition of imipenem further lowered the MIC_90_ of SD by one two-fold dilution.

**Conclusions:**

This study demonstrated the potential utility of SD for the treatment of infections caused by *A. baumannii*. If its clinical efficacy is confirmed, SD may be an important therapeutic option for CRAB infections.

## Introduction

Infections caused by multidrug-resistant (MDR) *A. baumannii* pose a serious threat to global health. These infections include ventilator-associated pneumonia, bacteremia, complicated urinary tract infections and skin and soft tissue infections, in both healthy and immuno-compromised individuals (Lee et al., 2017). The mortality rates of these infections reach 33%, and carbapenem resistance was associated with a greater risk of death (pooled odds ratio 2.22, 95% CI 1.66-2.98) [European Centre for Disease Prevention and Control (ECDC), 2013; Lemos et al., 2014]. A recent analysis of the global prevalence of antibiotic resistance in *A. baumannii* infections found a prevalence of resistance to imipenem of 73.9–77.8%, which represented a dramatic increase since 2005 (Xie et al., 2018).

The epidemiological status of carbapenem-resistant *A. baumannii* (CRAB) in Greece is defined as endemic situation, with most hospitals repeatedly facing cases admitted from endemic sources [European Centre for Disease Prevention and Control (ECDC), 2013]. The resistance to carbapenems in *A. baumannii* precludes the use of β-lactam therapies; the main mechanism responsible is the production of oxacillinases (class D β-lactamases), with the most frequent ones being OXA-23, OXA-24/40 and OXA-58 (D'Arezzo et al., 2010; Gogou et al., 2011; Schleicher et al., 2013). Among the limited β-lactams that retain a degree of activity against *A. baumannii* is sulbactam (SUL), a semi-synthetic penicillanic acid, which is a first-generation β-lactamase inhibitor, with limited activity against class A serine β-lactamases (Seifert et al., 2020; Shapiro et al., 2021). SUL inhibits *A. baumannii* enzymes that are involved in bacterial peptidoglycan synthesis, such as PBP1a, PBP1b and PBP3 (Higgins et al., 2014). However, due to its susceptibility to degradation by a variety of acquired or upregulated β-lactamases, SUL has limited clinical utility against infections caused by CRAB (Penwell et al., 2015).

A promising new therapeutic option for CRAB is the combination of sulbactam with durlobactam (SD), a new member of the diazabicyclooctane class of β-lactamase inhibitors, with broad spectrum activity against Ambler class A, C and D serine β-lactamases, resulting in the restoration of the susceptibility of CRAB isolates to β-lactams (Durand-Réville et al., 2017). Durlobactam (DUR) restores SUL susceptibility of *A. baumannii* strains overexpressing individual β-lactamases (Seifert et al., 2020; Shapiro et al., 2021) as well as diverse clinical isolates, in international, contemporary surveillance studies (McLeod et al., 2020; Yang et al. 2020; Nodari et al. 2021). SD is currently in late-stage development for the treatment of infections caused by *Acinetobacter* spp.

The aim of this study was to examine the *in vitro* potency of the SD combination on a collection of previously characterized, non-duplicate isolates of CRAB from Greece harboring acquired β-lactamases.

## Materials And Methods

### Bacterial Strains

The study included 190 non-repetitive CRAB isolates recovered during 2015 from 11 geographically distinct tertiary hospitals, located throughout Greece and selected during a previous nationwide study randomly from a collection of 2,500 *A. baumannii* isolates. The clinical samples included blood, bronchial aspirates, urine, superficial or deep tissue wounds, peritoneal and pleural effusions, cerebrospinal fluids and intra-abdominal secretions. All isolates were previously characterized and confirmed to be *A. baumannii* by PCR/sequencing for the intrinsic *bla*_OXA-51-like_ gene (Pournaras et al., 2017). In particular, clonality was tested by a scheme based on two multiplex PCRs, that selectively amplified alleles of the *ompA*, *csuE* and *bla*_OXA-51-like_ (Turton et al., 2007; Giannouli et al., 2010) and single-locus *bla*_OXA-51-like_ sequence-based typing (SBT) (Pournaras et al., 2014). The SBT assigned 153 isolates (80.5%) to IC2, 36 isolates to IC1 (18.9%) and 1 isolate to G6 (0.5%). Of the 153 IC2 isolates, all had *bla*_OXA-23-like_ and three had both *bla*_OXA-23-like_ and *bla*_OXA-58-like_. As far as IC1 clone, 34 isolates (94.5%) had *bla*_OXA-23-like_ and two (5.5%) had *bla*_OXA-58-like_. As for the β-lactamases carried by the study isolates, the *bla*_OXA-23-like_ gene was identified in 187 isolates (98.4%), *bla*_OXA-23-like_ together with *bla*_OXA-58-like_ in three (1.6%), *bla*_OXA-58-like_ in two (1.0%) and *bla*_OXA-40-like_ in one isolate (0.5%).

### Antimicrobial Susceptibility

Antimicrobial susceptibility was determined using broth microdilution in freshly prepared cation-adjusted Mueller–Hinton broth (CAMHB) following CLSI recommendations (CLSI, 2018; CLSI, 2019). Pre-manufactured, frozen 96-well plates containing 50 μL of 2x antimicrobial drug concentrations were supplied by Entasis Therapeutics. The 190 CRAB isolates were tested against SUL, amikacin (AMK), minocycline (MIN), imipenem (IMP), meropenem (MER), colistin (COL), SD and IMP combined with SD (SID). The concentration ranges tested in 2-fold dilutions were for SUL, 0.06 to 64 µg/ml; SD [durlobactam (DUR) fixed at 4 µg/ml], 0.06/4 to 64/4 µg/ml; SID (1/1/2 ratio), 0.06/0.06/0.12 to 64/64/128; AMK, 0.12 to 128 µg/ml; COL, 0.06 to 64 µg/ml; IMP, 0.06 to 64 µg/ml; MER, 0.06 to 64 µg/ml; and MIN, 0.03 to 32 µg/ml. The combination of SUL, IMP and DUR was tested in a fixed 1:1:2 ratio titrated in 2-fold dilutions. MICs were interpreted according to CLSI guidelines and susceptibilities were determined using CLSI breakpoints, where applicable. Each experiment included testing of CLSI-approved quality control organisms NCTC 13304 (*A. baumannii*), ATCC 25922 (*Escherichia coli*) and ATCC 27853 (*Pseudomonas aeruginosa).* The minimal inhibitory concentration (MIC) of each antibiotic was determined by visual inspection for each strain after incubation for 20 hours at 35°C.

### Next Generation Sequencing of Isolates With Elevated SD MICs

Three isolates with SD MICs >8/4 µg/ml were analyzed by next Generation Sequencing. Genomic DNA was purified from the isolates using a Sigma-Aldrich GenElute bacterial genomic DNA kit. Genomic libraries were assembled using a Nextera XT library preparation kit and sequenced using an Illumina MiSeq system with 300-bp paired-end reads and a coverage of ≥ 50X. Assembly and analysis of the whole genome sequencing was performed using CLC Genomics Workbench v21.0.3 (Qiagen, Germantown, MD). Paired Fastq files were processed and analyzed as follows: raw reads were trimmed of any remaining barcode sequences as well as trimmed for quality. Reads were then *de novo* assembled using fraction length=0.8 and similarity fraction=0.9 using default mismatch/insertion/deletion costs. Consensus sequences were extracted and contigs greater than 500bp were assembled. B-lactamase genes for each strain were identified by BLAST against a local β-lactamase database. Additionally, mutations in efflux, permeation, and PBP proteins were identified by BLAST analysis against the *A. baumannii* ATCC 17978 reference strain. The genomic sequences of the three strains tested were deposited at http://www.ncbi.nlm.nih.gov/bioproject/781741.

## Results

The SD MIC_50/90_ values were 4/4 and 8/4 µg/ml, respectively. The SID MIC_50/90_ values were 2/2/4 and 4/4/8 µg/ml, respectively (**Table 1**). The MIC_50/90_ values of currently-used comparator antimicrobials were: SUL (64/>64), COL (2/16), MIN (16/32), IMP (>64/>64), MER (>64/>64), and AMK (>128/>128) µg/ml (**Table 2**).

**Table 1:** MIC distribution of the 190 CRAB isolates for SUL, SD and SID and their MIC_50_, MIC_90_ values.

MIC (μg/ml)											
**Antimicrobial agent**	**0.5**	**1**	**2**	**4**	**8**	**16**	**32**	**64**	**>64**	**MIC_50_**	**MIC_90_**
**SUL**					3	11	82	77	17	64	>64
**SD** **(Durlobactam fixed** **at 4 μg/ml)**	2	20	61	84	20	2			1	4	8
**SID (1:1:2)**	2	19	84	83	1			1		2	4

**Table 2:** Resistance rates of the 190 CRAB isolates and their international clonal lineages IC1 and IC2 for SUL, COL, IMP, MER, AMK, MIN, SD and SID and their MIC_50_, MIC_90_ values.

Antimicrobial agent	OVERALL (n = 190)					IC1 (n = 36)					IC2 (n = 153)				
	MIC_50_	MIC_90_	S %	I %	R %	MIC_50_	MIC_90_	S %	I %	R %	MIC_50_	MIC_90_	S %	I %	R %
**SUL**	64	>64	–		–	32	64	–		–	64	>64	–		–
**COL**	2	16	67.9	0	32.1	1	16	75	0	25	2	16	66	0	34
**IMP**	>64	>64	0	0	100	64	>64	0	0	100	>64	>64	0	0	100
**MER**	>64	>64	0	0	100	64	>64	0	0	100	>64	>64	0	0	100
**AMK**	>128	>128	2.1	0.5	97.4	>128	>128	2,8	0	97,2	>128	>128	2	0,7	97,3
**MIN**	16	32	25.3	17.4	57.3	2	4	97,2	0	2,8	16	32	7,8	21	71,2
**SD (Durlobactam fixed at 4 μg/ml)**	4	8	–		–	4	4	–		–	4	4	–		–
**SID (1:1:2)**	2	4	–		–	2	4				2	4				

All isolates had IMP and MER MICs ≥32 μg/ml. Resistance rates to comparators were as follows: IMP, 100%; MER, 100%; AMK, 97.4%; and MIN, 57.3%. Of concern, 61 of the 190 (32.1%) isolates were resistant to COL and among isolates from blood cultures, the resistance rate reached 36%. Among the COL-resistant isolates, 54 (88.5%) had low SD MICs of ≤4 μg/ml and all (100%) had SID MICs of ≤4/4/8 µg/ml.

Of the isolates, 87.9% had SD MICs of ≤4/4 µg/ml and only three isolates had SD MIC >8 µg/ml. These three isolates were submitted to NGS sequencing. The genomic characteristics of these isolates are shown in **Table 3**. In brief, they belonged to three different MLST types (ST-1834, SD MIC 16 μg/ml; ST-1294 SD MIC >64 μg/ml; and ST-425, SD MIC 16 μg/ml). They all carried *bla*_OXA-23_ and *bla*_OXA-66_ and all encoded the same A515V variant of the PBP3 gene that likely confers resistance to SUL, considering its proximity to the SUL binding site (Papp-Wallace et al., 2012). The SID MIC for both ST-1834 and ST-425 isolates was 4 μg/ml (i.e. addition of IMP helped reduce the SD MIC), while the ST-1294 isolate had SD MIC of > 64 μg/ml and SID MIC 64/64/128 μg/ml. The latter isolate also harbored the NDM metallo-β-lactamase gene, which DUR does not inhibit.

**Table 3:** Accession numbers and resistance mechanisms detected by next generation sequencing of the three isolates with SD MICs > 8 μg/ml.

Genome accession	SD MICs (μg/ml)	SID MICs (μg/ml)	MLST Classification	Encoded *BLAs*				Other mutations	
				Class A	Class B	Class C	Class D	PBPs	efflux components
JAJKGX000000000	16	4/4/8	ST-1834, 436/PST-2	TEM-1	–	ADC-73	OXA-23; OXA-66	PBP3 [A515V]	–
JAJKGW000000000	>64	64/64/128	ST-1294/PST-570	TEM-1	NDM-1	ADC-73	OXA-23; OXA-66	PBP3 [A515V]	–
JAJKGV000000000	16	4/4/8	ST-425/PST-2	–	–	ADC-188	OXA-23; OXA-66	PBP3 [A515V]	AdeR [G25S]

## Discussion

The most prevalent mechanism of carbapenem resistance among *A. baumannii* is associated with carbapenem-hydrolysing enzymes that belong to Ambler class D and B β-lactamases (Jeon et al., 2015; Lee et al., 2017; Wong et al., 2017; Lötsch et al., 2020). The rapid rise of carbapenem resistance among *A. baumannii* isolates limits the available therapeutic options and poses a serious need for new antimicrobial agents. In particular, according to 2019 data from the European Antimicrobial Resistance Surveillance Network (EARS-Net), nearly a third of invasive *Acinetobacter* spp. isolates in the EU/EEA are already resistant to carbapenems [European Centre for Disease Prevention and Control (ECDC), 2019; Lötsch et al., 2020]. Carbapenem resistance rates are higher than 50% in southern and eastern European countries [European Centre for Disease Prevention and Control (ECDC), 2019], while in Greece, according to data from the Hellenic CDC, IMP resistance rates are currently exceeding 90% (WHONET). Of note, in our representative countrywide collection, CRAB isolates exhibited particularly high levels of resistance to last-line antimicrobials in addition to carbapenems, including AMK, SUL, MIN and COL.

Among the few therapeutic options that show efficacy against CRAB isolates is COL, used both as monotherapy or in combination with other antimicrobials. Still, while COL is a key drug, there are concerns, not only about its toxicity profiles, but also its rising resistance rates (Viehman et al., 2014). A meta-analysis on the prevalence of *A. baumanni* antimicrobial resistance worldwide from 2000 to 2017, showed that the overall global resistance rate reaches 11.2% (Pormohammad et al., 2020). Herein, COL showed a much higher resistance rate of 32.1% and among isolates, collected from blood cultures, the rate reached 36%.

The carbapenem resistance problem of *A. baumannii* can be overcome by the use of expanded-spectrum serine β-lactamase inhibitors, which may inhibit class A, C or D β-lactamases, resulting in restoration of β-lactam activity. SD is a promising combination and its spectrum of activity can address MDR *A. baumannii.* In our study, the addition of DUR at a fixed concentration of 4 µg/ml lowered the MIC_50_ and MIC_90_ of SUL from 64 and >64 to 4 and 8 μg/ml, respectively, except for the isolate that encoded metallo-β-lactamase. The MIC_90_ of SD was considerably lower than the MIC_90_ of carbapenems, MIN and AMK and also lower from that of COL [16 μg/ml]. 88.5% of non-susceptible to COL isolates had SD MICs ≤ 4 μg/ml.

*A. baumannii* isolates belonging to IC1 showed generally more sensitive profiles compared to IC2, concerning SUL, COL and carbapenems. For MIN, the resistance rates between IC1 and IC2 isolates were 2.8% and 71.2%, respectively. There was no difference on the MICs of SD and SID among the isolates of the two clonal lineages. For both IC2 and IC2 isolates, MIC_50/90_ of SD and SID were at 4/4 and 2/4, respectively.

Compared to other studies on the activity of SD, our collection of isolates presented higher resistance rates and MICs for the comparators, as well as for SD. In particular, both the SD MIC_50_ and MIC_90_ of the strains in the present study exceeded by one to three-fold the respective values of those reported in international studies (McLeod et al., 2020; Seifert et al., 2020; Yang et al., 2020; Nodari et al., 2021), indicating the presence of less susceptible strains in Greek hospitals. COL MICs were also considerably lower in three of those studies (MIC_90_ 1 μg/ml in references Higgins et al., 2004; Pournaras et al., 2017; Seifert 2020), with only one study from China reporting COL MIC_90_ 128 μg/ml (Durand-Réville et al., 2017). Interestingly, the addition of IMP to SD lowered its MIC90 by one two-fold dilution.

Our study clearly showed that SD had excellent *in vitro* activity against CRAB isolates that were highly resistant to IMP, MER, AMK, MIN and COL. In addition, SD showed favorable clinical efficacy and safety in a recently completed, global phase 3 study (Entasis Therapeutics, 2019), https://investors.entasistx.com/news-releases/news-release-details/entasis-therapeutics-announces-positive-topline-results). If approved this combination may provide an important therapeutic option for infections due to MDR *A. baumannii*, including CRAB.

Part of this work was presented at the 31st European Congress of Clinical Microbiology and Infectious Diseases (ECCMID), Online, 9–12 July 2021.

## Data Availabilty Statement

The raw data supporting the conclusions of this article will be made available by the authors, without undue reservation.

## Author Contributions

All authors listed have made a substantial, direct and intellectual contribution to the work, and approved it for publication.

## Funding

The research being submitted received a grant from ENTASIS Therapeutics. This grant did not include open access publication fees, which will be paid by the research account of our Laboratory in our institution (National and Kapodistrian University of Athens Special Account for Research Grants).

## Conflict of Interest

This study received funding from Entasis Therapeutics. The funder had the following involvement with the study: provision of MIC plates and next generation sequencing analysis. Entasis was not involved in the collection, analysis, interpretation of susceptibility data, the writing of this article or the decision to submit it for publication. All authors declare no other competing interests.

## Publisher’s Note

All claims expressed in this article are solely those of the authors and do not necessarily represent those of their affiliated organizations, or those of the publisher, the editors and the reviewers. Any product that may be evaluated in this article, or claim that may be made by its manufacturer, is not guaranteed or endorsed by the publisher.

## References

[B1] CLSI. (2018). Methods for Dilution Antimicrobial Susceptibility Tests for Bacteria That Grow Aerobically, Eleventh Edition. CLSI standard M07. Wayne, PA: Clinical and Laboratory Standards Institute.

[B2] CLSI. (2019). Performance Standards for Antimicrobial Susceptibility Testing, Twenty-Ninth Edition. CLSI supplement M100. Wayne, PA: Clinical and Laboratory Standards Institute.

[B3] D'Arezzo S. Principe L. Capone A. Petrosillo N. Petrucca A. Visca P. (2010). Changing Carbapenemase Gene Pattern in an Epidemic Multidrug-Resistant *Acinetobacter Baumannii* Lineage Causing Multiple Outbreaks in Central Italy. J. Antimicrob. Chemother. 66, 54–61. doi: 10.1093/jac/dkq407 21088019 PMC3031335

[B4] Durand-Réville T. Guler S. Comita-Prevoir J. Chen B. Bifulco N. Huynh H.. (2017). ETX2514 Is a Broad-Spectrum β-Lactamase Inhibitor for the Treatment of Drug-Resistant Gram-Negative Bacteria Including Acinetobacter Baumannii. Nat. Microbiol. 2, 17104. doi: 10.1038/nmicrobiol.2017.104 28665414

[B5] Entasis Therapeutics. (2019). Study to Evaluate the Efficacy and Safety of Intravenous Sulbactam-ETX2514 in the Treatment of Patients With Infections Caused by Acinetobacter Baumannii-Calcoaceticus Complex (ATTACK). Available at: https://clinicaltrials.gov/ct2/show/NCT03894046?term=sulbactam&draw=4&rank=21.

[B6] European Centre for Disease Prevention and Control (ECDC). (2013). Carbapenemase-Producing Bacteria in Europe - Interim Results From the European Survey on Carbapenemase-Producing Enterobacteriaceae (EuSCAPE) Project 2013 (Stockholm: ECDC). Available at: https://ecdc.europa.eu/sites/portal/files/media/en/publications/Publications/antimicrobial-resistance-carbapenemase-producing-bacteria-europe.

[B7] European Centre for Disease Prevention and Control (ECDC). (2019). Surveillance of Antimicrobial Resistance in Europe 2018. Annual Report of the European Antimicrobial Resistance Surveillance Network (EARS-Net) (Stockholm: ECDC). Available at: https://www.ecdc.europa.eu/sites/default/files/documents/surveillance-antimicrobial-resistance-Europe-2018.

[B8] Giannouli M. Cuccurullo S. Crivaro V. Di Popolo A. Bernardo M. Tomasone F.. (2010). Molecular Epidemiology of Multidrug-Resistant Acinetobacter Baumannii in a Tertiary Care Hospital in Naples, Italy, Shows the Emergence of a Novel Epidemic Clone. J. Clin. Microbiol. 48, 1223–1230. doi: 10.1128/JCM.02263-09 20181918 PMC2849555

[B9] Gogou V. Pournaras S. Giannouli M. Voulgari E. Piperaki E. Zarrilli R.. (2011). Evolution of Multidrug-Resistant Acinetobacter Baumannii Clonal Lineages: A 10 Year Study in Greece (2000-09). J. Antimicrob. Chemother. 66, 2767–2772. doi: 10.1093/jac/dkr390 21933784

[B10] Higgins P. Wisplinghoff H. Stefanik D. Seifert H. (2004). *In Vitro* Activities of the β-Lactamase Inhibitors Clavulanic Acid, Sulbactam, and Tazobactam Alone or in Combination With β-Lactams Against Epidemiologically Characterized Multidrug-Resistant *Acinetobacter Baumannii* Strains. Antimicrob. Agents Chemother. 48, 1586–1592. doi: 10.1128/AAC.48.5.1586-1592.2004 15105109 PMC400525

[B11] Jeon J. Lee J. Lee J. Park K. Karim A. Lee C.. (2015). Structural Basis for Carbapenem-Hydrolyzing Mechanisms of Carbapenemases Conferring Antibiotic Resistance. Int. J. Mol. Sci. 16, 9654–9692. doi: 10.3390/ijms16059654 25938965 PMC4463611

[B12] Lee C. Lee J. Park M. Park K. Bae I. Kim Y.. (2017). Biology of *Acinetobacter Baumannii*: Pathogenesis, Antibiotic Resistance Mechanisms, and Prospective Treatment Options. Front. Cell. Infect. Microbiol. 7, 55. doi: 10.3389/fcimb.2017.00055 28348979 PMC5346588

[B13] Lemos E. de la Hoz F. Einarson T. McGhan W. Quevedo E. Castañeda C.. (2014). Carbapenem Resistance and Mortality in Patients With Acinetobacter Baumannii Infection: Systematic Review and Meta-Analysis. Clin. Microbiol. Infect. 20, 416–423. doi: 10.1111/1469-0691.12363 24131374

[B14] Lötsch F. Albiger B. Monnet D. Struelens M. Seifert H. Kohlenberg A. (2020). Epidemiological Situation, Laboratory Capacity and Preparedness for Carbapenem-Resistant Acinetobacter Baumannii in Europe, 2019. Eurosurveillance 25, 2001735. doi: 10.2807/1560-7917.ES.2020.25.45.2001735 PMC7667627 33183407

[B15] McLeod S. Moussa S. Hackel M. Miller A. (2020). *In Vitro* Activity of Sulbactam-Durlobactam Against *Acinetobacter Baumannii-Calcoaceticus* Complex Isolates Collected Globally in 2016 and 2017. Antimicrob. Agents Chemother 64, e02354–19. doi: 10.1128/AAC.02534-19 PMC7179289 31988095

[B16] Nodari C. S. Santos F. F. Kurihara M. N. L. Valiatti T. B. Cayô R. Gales A. C. (2021). In Vitro Activity of Sulbactam/Durlobactam Against Extensively Drug-Resistant Acinetobacter baumannii Isolates Belonging to South American Major Clones. J. Glob. Antimicrob. Resist. 25, 363–366. doi: 10.1016/j.jgar.2021.05.001 33989848

[B17] Papp-Wallace K. M. Senkfor B. Gatta J. Chai W. Taracila M. A. Shanmugasundaram V.. (2012). Early Insights Into the Interactions of Different β-Lactam Antibiotics and β-Lactamase Inhibitors Against Soluble Forms of *Acinetobacter Baumannii* PBP1a and *Acinetobacter* Sp. PBP3. Antimicrob. Agents Chemother. 56, 5687–5692. doi: 10.1128/AAC.01027-12 22908165 PMC3486531

[B18] Penwell W. F. Shapiro A. B. Giacobbe R. A. Gu R. F. Gao N. Thresher J.. (2015). Molecular Mechanisms of Sulbactam Antibacterial Activity and Resistance Determinants in Acinetobacter baumannii. Antimicrob. Agents Chemother. 59, 1680–1689. doi: 10.1128/AAC.04808-14 25561334 PMC4325763

[B19] Pormohammad A. Mehdinejadiani K. Gholizadeh P. Nasiri M. Mohtavinejad N. Dadashi M.. (2020). Global Prevalence of Colistin Resistance in Clinical Isolates of Acinetobacter Baumannii: A Systematic Review and Meta-Analysis. Microb. Pathogenesis 139, p.103887. doi: 10.1016/j.micpath.2019.103887 31765766

[B20] Pournaras S. Dafopoulou K. Del Franco M. Zarkotou O. Dimitroulia E. Protonotariou E.. (2017). Predominance of International Clone 2 OXA-23-Producing-*Acinetobacter Baumannii* Clinical Isolates in Greece, 2015: Results of a Nationwide Study. Int. J. Antimicrob. Agents 49, 749–753. doi: 10.1016/j.ijantimicag.2017.01.028 28427842

[B21] Pournaras S. Gogou V. Giannouli M. Dimitroulia E. Dafopoulou K. Tsakris A.. (2014). Single-Locus-Sequence-Based Typing of blaOXA-51-Like Genes for Rapid Assignment of Acinetobacter Baumannii Clinical Isolates to International Clonal Lineages. J. Clin. Microbiol. 52, 1653–1657. doi: 10.1128/JCM.03565-13 24622099 PMC3993655

[B22] Schleicher X. Higgins P. Wisplinghoff H. Körber-Irrgang B. Kresken M. Seifert H. (2013). Molecular Epidemiology of *Acinetobacter Baumannii* and *Acinetobacter Nosocomialis* in Germany Over a 5-Year Period (2005–2009). Clin. Microbiol. Infect 19, 737–742. doi: 10.1111/1469-0691.12026 23034071

[B23] Seifert H. Müller C. Stefanik D. Higgins P. Miller A. Kresken M. (2020). *In Vitro* Activity of Sulbactam/Durlobactam Against Global Isolates of Carbapenem-Resistant Acinetobacter Baumannii. J. Antimicrob. Chemother. 75, 2616–2621. doi: 10.1093/jac/dkaa208 32516359

[B24] Seifert H. Stefanik D. Olesky M. Higgins P. (2020). *In Vitro* Activity of the Novel Fluorocycline TP-6076 Against Carbapenem-Resistant Acinetobacter Baumannii. Int. J. Antimicrob. Agents 55, 105829. doi: 10.1016/j.ijantimicag.2019.10.010 31669740

[B25] Shapiro A. Moussa S. McLeod S. Durand-Réville T. Miller A. (2021). Durlobactam, a New Diazabicyclooctane β-Lactamase Inhibitor for the Treatment of *Acinetobacter* Infections in Combination With Sulbactam. Front. Microbiol. 12, 709974. doi: 10.3389/fmicb.2021.709974 34349751 PMC8328114

[B26] Turton J. F. Gabriel S. N. Valderrey C. Kaufmann M. E. Pitt T. L. (2007). Use of Sequence-Based Typing and Multiplex PCR to Identify Clonal Lineages of Outbreak Strains of Acinetobacter Baumannii. Clin. Microbiol. Infect. 13, 807–815. doi: 10.1111/j.1469-0691.2007.01759.x 17610600

[B27] Viehman J. Nguyen M. Doi Y. (2014). Treatment Options for Carbapenem-Resistant and Extensively Drug-Resistant Acinetobacter Baumannii Infections. Drugs 74, pp.1315–1333. doi: 10.1007/s40265-014-0267-8 PMC4258832 25091170

[B28] WHONET (Greece). Available at: http://www.mednet.gr/whonet/.

[B29] Wong D. Nielsen T. Bonomo R. Pantapalangkoor P. Luna B. Spellberg B. (2017). Clinical and Pathophysiological Overview of *Acinetobacter* Infections: A Century of Challenges. Clin. Microbiol. Rev. 30, 409–447. doi: 10.1128/cmr.00058-16 27974412 PMC5217799

[B30] Xie R. Zhang X. Zhao Q. Peng B. Zheng J. (2018). Analysis of Global Prevalence of Antibiotic Resistance in *Acinetobacter Baumannii* Infections Disclosed a Faster Increase in OECD Countries. Emerging Microbes Infect 7, 1–10. doi: 10.1038/s41426-018-0038-9 PMC5849731 29535298

[B31] Yang Q. Xu Y. Jia P. Zhu Y. Zhang J. Zhang G.. (2020). In Vitro Activity of Sulbactam/Durlobactam Against Clinical Isolates of Acinetobacter baumannii Collected in China. J. Antimicrob. Chemother. 75, 1833–1839. doi: 10.1093/jac/dkaa119 32306049

